# Transient and steady-state auditory gamma-band responses in first-degree relatives of people with autism spectrum disorder

**DOI:** 10.1186/2040-2392-2-11

**Published:** 2011-07-05

**Authors:** Donald C Rojas, Peter D Teale, Keeran Maharajh, Eugene Kronberg, Katie Youngpeter, Lisa B Wilson, Alissa Wallace, Susan Hepburn

**Affiliations:** 1 Department of Psychiatry, University of Colorado Denver, Aurora, CO, 80241, USA

## Abstract

**Background:**

Stimulus-related γ-band oscillations, which may be related to perceptual binding, are reduced in people with autism spectrum disorders (ASD). The purpose of this study was to examine auditory transient and steady-state γ-band findings in first-degree relatives of people with ASD to assess the potential familiality of these findings in ASD.

**Methods:**

Magnetoencephalography (MEG) recordings in 21 parents who had a child with an autism spectrum disorder (pASD) and 20 healthy adult control subjects (HC) were obtained. Gamma-band phase locking factor (PLF), and evoked and induced power to 32, 40 and 48 Hz amplitude-modulated sounds were measured for transient and steady-state responses. Participants were also tested on a number of behavioral and cognitive assessments related to the broad autism phenotype (BAP).

**Results:**

Reliable group differences were seen primarily for steady-state responses. In the left hemisphere, pASD subjects exhibited lower phase-locked steady-state power in all three conditions. Total γ-band power, including the non-phase-locked component, was also reduced in the pASD group. In addition, pASD subjects had significantly lower PLF than the HC group. Correlations were seen between MEG measures and BAP measures.

**Conclusions:**

The reduction in steady-state γ-band responses in the pASD group is consistent with previous results for children with ASD. Steady-state responses may be more sensitive than transient responses to phase-locking errors in ASD. Together with the lower PLF and phase-locked power in first-degree relatives, correlations between γ-band measures and behavioral measures relevant to the BAP highlight the potential of γ-band deficits as a potential new autism endophenotype.

## Background

Autism spectrum disorders (ASD) are clinically defined by impairments in social interaction and communication and by restricted/stereotyped behaviors. The prevalence for ASD, which includes autistic disorder, Asperger's syndrome and pervasive developmental disorder - not otherwise specified, is estimated to be as high as 1 in 110 [CDC, [1]]. Although diagnosable medical conditions, including genetic syndromes, are estimated to account for as many as 10% of cases, most cases remain idiopathic [2,3]. Family studies indicate that idiopathic ASD is highly heritable [4,5], with an estimated heritability as high as 90%. Studies of first-degree relatives have shown increased prevalence of anxiety and depression, personality changes, deficits in the social use of language (that is, pragmatics) and significantly higher scores on assessments of autism traits such as social responsiveness [6-10]. This subclinical expression of the ASD phenotype is termed the broad autism phenotype (BAP) and provides further evidence for the heritability of autism.

Although studies of first-degree relatives have identified a broad range of changes in the behavioral phenotype, few studies aside from genetics have examined the underlying biology of the BAP. However, studies of parents and siblings of people with ASD have reported increased rates of macrocephaly [11,12], enlarged hippocampi [13], cortical gray-matter changes [14], altered occulomotor function [15,16], increased platelet serotonin levels [17], reductions in face-specific early visual processing [18], and reduced γ-band oscillatory phase-locking [19].

Gamma-band oscillatory activity (that is, 30 to 80 Hz) is of significant interest as a biomarker and/or endophenotype in ASD for two reasons: 1) there is a putative relationship between perceptual binding and/or connectivity and γ [20,21], which have been proposed as cognitive deficits in the disorder [22-25]; and 2) mechanisms for generating γ-band activity in the cerebral cortex and hippocampus are relatively well characterized [26].

Auditory γ-band responses are not unitary, however. Early, obligatory γ-band responses are produced to any auditory stimulus, and usually appear at 30 to 80 milliseconds after stimulus [27]. This early, highly phase-locked response is called the transient γ-band response (tGBR). However, when stimuli are modulated in amplitude, either as part of a train of clicks or by formal amplitude modulation, a later auditory steady-state response (ASSR) is produced, in this case at or near the frequency of modulation, which peaks at rates in the γ-band range [28,29]. Steady-state stimulation produces both types of responses [30]. The mechanisms of generation for these two types of responses, and also whether they are related to cognitive or perceptual processes, may vary. The ASSR may partly reflect a linear superposition of transient mid-latency auditory evoked responses [31,32], although this is not completely accepted by all investigators [28,30,33,34]. Regardless, the purported association between cognitive functions and the tGBR is not established for the ASSR.

We first reported a significant reduction in MEG-measured evoked or phase-locked ASSR power in children and adolescents with autism compared with control subjects matched for age and gender [35]. Subsequently, we found that adults with ASD and first-degree relatives of people with ASD exhibited reduced tGBR evoked power and increased tGBR induced power, compared with healthy controls [19]. Across trials evoked responses are consistently phase-locked to the stimulus, whereas induced responses are not. Together, these two types of responses constitute total stimulus-related power. Increases in non-phase-locked γ-band power have also been reported in other studies [36-38]. We proposed that the deficit in the γ-band electrophysiology may in reduced inter-trial phase-locking to the stimulus, which causes a shift in γ-band power from phase-locked, evoked power to non-phase-locked induced power, while preserving total γ-band power. A recent MEG study replicated reduced auditory tGBR phase-locking in a sample of children with ASD [39], but did not report significant differences in either evoked or induced power. It should be noted that phase-locking factor (PLF), also known as inter-trial coherence, is an amplitude-independent measure, unlike evoked power, so although the two measures may be correlated, phase-locking will tend to be more robust in noisy data, having lower between- and within-subject variance [40] (see Additional file 1).

Our previous report on parents of children with ASD measured only the tGBR component [19], whereas our original finding of reduced evoked γ-band power in children with ASD reflected only the ASSR component [35]. The current study was therefore designed to ascertain whether adult first-degree relatives of people with autism exhibit changes in both the tGBR and ASSR. We hypothesized that phase-locked auditory evoked γ-band activity, as well as being a direct measure of stimulus-related phase locking, would be lower in first-degree relatives of people with ASD for both types of γ-band responses. Three different amplitude modulation rates were used to assess whether relatives of people with ASD would exhibit changes in ASSR-evoked γ-band activity specific to 40 Hz or across a wider γ-band range. Measures associated with the BAP, including the Autism-Spectrum Quotient (AQ) [41] and the Social Responsiveness Scale (SRS)[42], were included to assess potential relationships between the BAP and γ-band activity, because neither of our earlier studies obtained such measures for correlation with the electrophysiological data.

## Results

### Sample characteristics

No significant differences in age, gender distribution, socioeconomic status or general cognitive ability were present between the two groups. With respect to cognitive and autism spectrum measures, only the local details sub-score of the AQ differed significantly between groups (Table 1).

**Table 1:** Demographic and behavioural characteristics for participants.

	Control (N = 20)	Parent (N = 21)	T or χ2
Age	43.84 (6.86)	43.67 (7.33)	.08

Women/Men	13/6	15/6	.04

SES	42.58 (10.79)	41.33 (10.84)	.37

Verbal IQ	114.60 (11.82)	111.19 (9.00)	1.04

Performance IQ	116.40 (11.18)	113.43 (13.63)	.77

FSIQ	117.50 (11.98)	114.00 (13.36)	.96

AQ total	15.80 (6.35)	15.33 (5.56)	.25

AQ social skill	2.50 (1.91)	2.62 (2.18)	-.19

AQ attention switch	3.55 (2.40)	3.76 (2.10)	-.30

AQ local detail	5.85 (1.53)	4.57 (1.78)	2.47*

AQ communication	1.50 (1.73)	2.29 (1.52)	-1.54

AQ imagination	2.70 (1.90)	2.24 (1.78)	.80

SRS	38.33 (27.29)	38.90 (22.91)	-.55

Numbers in parentheses are standard deviations. * P < 0.05. Degrees of freedom = 39 for all t-tests. FSIQ = Full scale IQ. AQ = Autism-Spectrum Quotient. SRS = Social Responsiveness Scale. SES = Socioeconomic status [83].

### Dipole parameters

To examine group differences on dipole location, a 2 × 2 (group by hemisphere) multivariate analysis of variance (MANOVA) was used, with *x*, *y *and *z *locations as the dependent variables. The absolute value of the *x *coordinate was used in order to avoid artificially inflating the significance of the hemisphere effects because of the sign difference between left and right hemispheres. The main effects and interaction term were non-significant.

To assess dipole amplitude, Q_mag _(in units of nA-m, the square root of the sums of squared magnitudes for the dipole in *x*, *y *and *z *orientations) was evaluated using a 2 × 2 (group by hemisphere) mixed design ANOVA. No significant effects were found for Q_mag_.

Finally, for the overall goodness of fit of the dipole model, a 2 × 2 (group by hemisphere) mixed model ANOVA was calculated. Only the hemisphere main effect was significant, *F*_(1, 39) _= 8.05, *P *< 0.01, indicating that the left hemisphere (0.984 ± 0.01) had a better fit than the right (0.975 ± .004).

### Transient γ-band responses

The tGBR PLF, evoked, induced and total power measures were entered as dependent measures into separate 2 × 2 × 3 (group by hemisphere by modulation frequency) mixed ANOVA designs.

**Figure 1:**
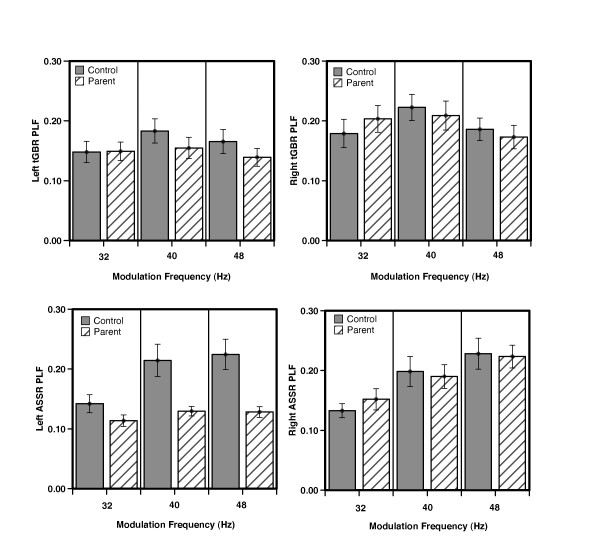
**Phase-locking factor results**. Phase-locking factor (PLF) group results (mean ± SE) for the left and right hemisphere dipole waveforms (shown in left and right columns respectively). Results for the transient and steady-state responses are shown in the top and bottom rows respectively.

**Figure 2:**
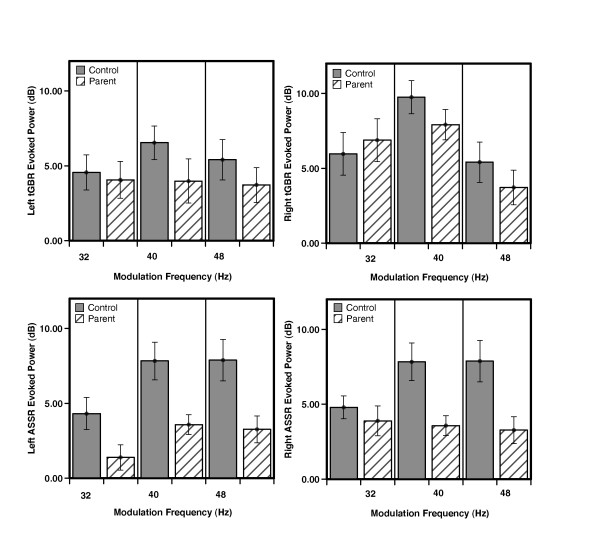
**Evoked power results**. Baseline normalized evoked amplitude group results (mean ± SE) for the left and right hemisphere dipole waveforms (shown in left and right columns respectively). Results for the transient and steady-state responses are shown in the top and bottom rows respectively.

For tGBR PLF, the only significant effect was a main effect of hemisphere, *F*_(1, 39) _= 8.03, *P *< 0.01, indicating slightly higher phase-locking in the right than in the left hemisphere across groups (see Figure 1). All other main effects and interaction terms were non-significant (all *P *> 0.10). As with PLF, for the baseline normalized evoked power, the main effect of hemisphere was the only significant effect, *F*_(1, 39) _= 9.06, *P *< 0.01, indicating that evoked power was higher in the right hemisphere than in the left (Figure 2). For baseline normalized induced power, the only significant effect was a main effect of hemisphere, *F*_(1, 39) _= 4.9, *P *< 0.03, indicating a greater reduction in induced power in the right hemisphere than in the left.

For total tGBR power, there was a significant group-by-frequency effect *F_(1, 39) _= 4.53, *P *< 0.05). Fisher's least significant difference (LSD) *post hoc *testing showed that although there were no differences in power between modulation frequencies for the HC group, the power of the tGBR response was significantly higher in the 32 Hz than in the 48 Hz condition for the pASD group (*P *= 0.04).

### Auditory steady-state responses

The ASSR PLF, evoked, induced and total power measures were also entered as dependent measures into separate 2 × 2 × 3 (group by hemisphere by modulation frequency) mixed ANOVA designs.

For ASSR PLF, there was a significant main effect of modulation frequency, *F*_(1, 39) _= 8.23, *P *< 0.001. *Post hoc *tests showed that the PLF for each modulation frequency differed from the other two frequencies (all *P *< 0.002), suggesting that ASSR PLF is strongly related to modulation frequency (see Figure 1). In addition, a significant hemisphere main effect, *F*_(1, 39) _= 5.27, *P *< 0.03, indicated that as with the tGBR, the ASSR PLF was higher in the right hemisphere. Although the group main effect was non-significant, *F*_(1, 39) _= 3.21, *P *= 0.08, there was a significant group-by-frequency interaction term, *F*_(1, 39) _= 4.66, *P *< 0.04. *Post hoc *tests showed that the groups differed significantly (HC > pASD) in the 48 Hz condition (*P *= 0.03), but not the 40 Hz (*P *= 0.07) or the 32 Hz conditions (*P *= 0.79). There was also a significant group-by-hemisphere interaction term (*F*_(1, 39) _= 4.18, *P *< 0.05), suggesting that the pASD group had lower PLF than the HC group in the left hemisphere (LSD *P *< 0.002), but not in the right (LSD *P *= 0.94). The three-way interaction term was non-significant.

The ASSR evoked power measure, like the ASSR PLF, also exhibited significant main effects of frequency (*F*_(1, 39) _= 44.76, *P *< 0.001) and hemisphere (*F*_(1, 39) _= 8.97, *P *< 0.01). *Post hoc *testing for the frequency main effect showed that the 32 Hz condition had significantly lower power than the 40 Hz (*P *< 0.001) and 48 Hz (*P *< 0.001) conditions. Unlike PLF, however, the main effect of group was significant (*F*_(1, 39) _= 5.14, *P *< 0.03), indicating lower evoked power in the pASD relative to the HC group across frequencies (Figure 2). The interaction terms for ASSR evoked power were all non-significant.

With ASSR induced power, there were significant main effects of hemisphere (*F*_(1, 39) _= 4.29, *P *< 0.05) and frequency (*F*_(1, 39) _= 15.06, *P *< 0.001). As with tGBR induced power, the reduction in ASSR induced power was greater in the right hemisphere. *Post hoc *testing on the frequency main effect indicated that the 40 and 48 Hz modulators had significantly greater induced power reductions than the 32 Hz condition (both *P *< 0.001).

For ASSR total power, there was a significant group-by-hemisphere interaction term, *F*_(1, 39) _= 4.67, *P *< 0.04, indicating that the pASD may have had lower power in the left but not right hemisphere compared with the HC group. However, the *post hoc *testing on groups within each hemisphere showed only a possible trend in the left hemisphere finding (LSD *P *< 0.07), and the right hemisphere comparison was non-significant (LSD *P *= 0.68).

### Baseline measure

Baseline power was compared using a group by hemisphere by modulation frequency (2 × 2 × 3) mixed design ANOVA. No significant main effects or interaction terms were seen (all *P *> 0.10).

### Time-frequency correlations

**Figure 3:**
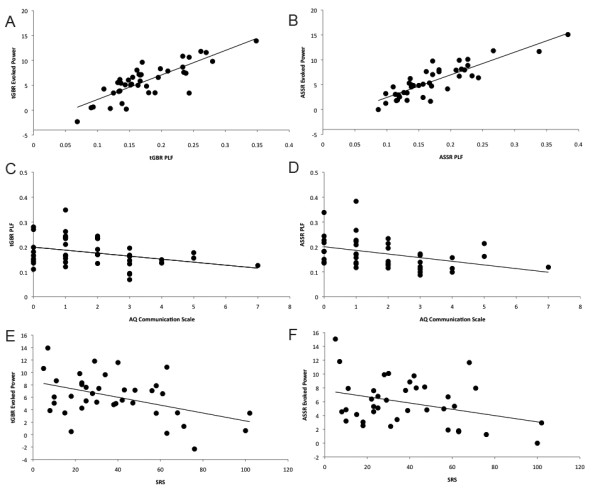
**Scatter plots of correlation results**. **(A)**Transient γ-band response (tGBR) phase-locking factor (PLF) and evoked power; **(B) **auditory steady-state response (ASSR) PLF and evoked power; **(C) **tGBR PLF and Autism-specturm Quotient (AQ) communication subscale; **(D) **ASSR PLF and AQ communication subscale; **(E) **tGBR evoked power and SRS; **(F) **ASSR evoked power and Social Responsiveness Scale (SRS). Black lines indicate linear regression line.

There was a significant correlation between mean phase-locked (evoked) tGBR γ-band power and mean tGBR PLF collapsed across group, condition and hemisphere (*r *= 0.81, *P *< 0.001). Similarly, mean ASSR γ-band phase-locked power was correlated with ASSR PLF, (*r *= 0.87, *P *< 0.001). Across groups, the AQ communication subscale score was inversely related to mean tGBR and ASSR PLF (*r *= -0.35, *P *< 0.05 and *r *= -0.39, *P *< 0.05). Mean tGBR and ASSR evoked power were also significantly negatively correlated with the SRS score (*r *= -0.45, *P *< 0.01 and *r *= -0.34, *P *< 0.05). No other correlations (mean tGBR and ASSR PLF and evoked power with Verbal IQ, performance IQ, AQ social skill, AQ local detail or AQ imagination) reached significance at α = 0.05. Figure 3 presents scatter plots of the significant correlations.

## Discussion

We found reduced ASSR γ-band evoked power and phase-locking in the pASD group relative to the HC group in the current study. This is consistent with our earlier published results for the auditory 40 Hz ASSR in children with autism, in which we reported reduced evoked power (PLF was not calculated in this earlier report) [35]. However, although mean tGBR evoked power and phase-locking were lower in the pASD group than in the HC group, the results for the tGBR portion of the response were not significant. This contrasts with our earlier findings for the transient γ-band responses, [19], which showed similar magnitude reductions for subjects with autism and pASD subjects compared to control subjects.

Several factors could contribute to the difference between the tGBR results of the current study and those of our previous study [19]. First, the stimuli in this study were amplitude-modulated specifically to produce robust ASSR responses, whereas the earlier study used pure tones, which only produce tGBR; however, we are not aware of any studies systematically comparing the effect of AM versus non-AM type stimuli on tGBR, so this is entirely speculative. Second, the pASD group in the earlier study may have had more BAP or relevant underlying physiological abnormalities than the group in the current study, which did not exhibit high ASD trait loading; however, we did not record such measures in the earlier study, preventing us from exploring this question. Finally, it should be noted that a more recent study in children with ASD did not find significant reductions in evoked power in the tGBR, although they did report significantly reduced PLF, which they attributed to the differential effects of noise on PLF versus evoked power [39] (see Additional file 1). It is therefore not clear even in probands with ASD that the finding of reduced evoked power is robust, and further studies with larger numbers of subjects (both probands and parents) will be needed to clarify these issues.

The current study also expanded on the earlier findings by including several modulation conditions, all of which produced some reduction in evoked power in both hemispheres. These reductions appeared stronger for the two highest modulation frequencies, but as we did not examine rates above 48 Hz, we do not know if there are higher rates at which the group differences are more pronounced. The restriction of the ASSR PLF findings to the left hemisphere is more consistent with our earlier published data on the ASSR in children with autism [35]. For tGBR elicited by pure-tone stimuli, our previous work suggested that reduced evoked power and PLF were present in both hemispheres [19]. This may represent a difference between ASSR and transient type auditory stimuli or γ-band response, although we did not replicate the earlier tGBR finding in this study. Recently, in a study of another set of potential ASD endophenotypes, Mosconi *et al*. [16] reported left-lateralized deficits in two measures of occulomotor function: open-loop pursuit gain and procedural learning for rightward saccades. Previous studies have suggested atypical lateralization of language-related brain structures in ASD [43-46]. Taken together, these findings may suggest abnormal cerebral lateralization and/or stronger left-hemisphere involvement in the disorder. Although group differences were noted primarily in the left-hemisphere data, across groups the tGBR and ASSR evoked power was stronger in the right hemisphere. This finding is consistent with previous EEG and MEG research [47-50]. Ross *et al*. [49] previously interpreted this in the context of right-hemisphere dominance for pitch perception, because the ASSR is known to closely entrain to the temporal envelope of sounds, and is very sensitive to disruptions in acoustic periodicity [51]. Previously, we have not found significant differences in total γ-band power (evoked plus induced), but for ASSR in the current study, there was a significant reduction in the left hemisphere of the pASD group. This will be important to parse in future studies, because if γ-band phase-locked power is significantly lower in ASD/pASD participants with no change in total γ-band power, it suggests a shift of γ-band activity from phase-locked to non-phase-locked activity in ASD, rather than a deficit in the generation of γ-band activity. Indeed, several previous studies have shown that induced (non-phase-locked) and spontaneous γ-band activities are increased in ASD [19,36-38]. If, however, there is a change in total stimulus-related power - in this case a reduction - it may suggest that γ-band-generating mechanisms themselves are altered, at least for auditory responses.

Gamma-band responses are of significant interest in ASD because their association with well-described cortical circuitry makes them ideal candidates for translational neuroscience. Glutamatergic input to inhibitory interneurons, particularly those expressing the calcium-binding protein parvalbumin (PV), results in the recurrent, phasic inhibitory modulation of pyramidal neurons [26,52-54]. PV-expressing interneurons play a crucial role in this inhibition via gamma-aminobutyric acid (GABA)_A_-receptor mediated mechanisms, the timing of which results in γ-band frequency output from the pyramidal neurons [55]. The GABA_A_-receptor antagonist bicuculline effectively eliminates γ-band oscillations [26]. Recently, studies of visual γ-band responses using MEG combined with magnetic resonance spectroscopy showed for the first time in human subjects that γ-band response frequency is associated with the cortical concentration level of GABA itself [56-58]. PV cell deficits are also a common feature across many mouse models of ASD [59]. In a potentially interesting parallel of our γ-band findings with two putative mouse models of ASD (prenatal exposure to valproic acid and neuroligin-3 R451C mutants), both models expressed deficits in PV inhibitory neurons in only one hemisphere [59].

The potential importance of GABA dysfunction to autism has been repeatedly stressed in the literature [60]. Blatt *et al*. [61] reported significantly reduced GABA_A_-receptor binding in strong binding regions of the hippocampus, with no significant differences noted in binding of serotonergic, cholinergic and glutamateric receptors. This has been extended recently to several areas of cortex and cerebellum [62]. A small study of children with autism aged 5 to 15 years reported increased plasma GABA levels [63], but the authors suggested that because the relationships between plasma, cerebrospinal fluid and brain levels of GABA are unknown, the implication of the finding for a specific central nervous system directionality is unclear. Messenger RNA levels of glutamate decarboxylase (GAD), the enzyme that converts glutamate to GABA and is closely related to intraneuronal GABA, have been reported to be reduced by about 40% in cerebellar Purkinje cells in people with autism [64], and up to 50% in parietal and/or cerebellar tissues, depending on the specific isomer (GAD65 or GAD67) [65].

A reduction in GAD expression also implies a corresponding increase in cortical glutamate levels. Increased glutamate concentrations have been reported in the hippocampal region of people with autism using proton magnetic resonance spectroscopy [66], consistent with previous reports of increased serum levels of glutamate [67], giving rise to a hyperglutamate hypothesis of autism [68], which suggests in part that higher glutamate levels in autism may be due to reductions in GAD. Alteration in the balance of cortical excitation and inhibition is the key prediction made by Rubenstein and Merzenich in their theoretical model of autism [69]. We propose that the γ-band phase-locking deficit seen in people with ASD and their first-degree relatives is a potential non-invasive biomarker reflective of this change in the excitation/inhibition balance.

The observed correlation between PLF and the AQ communication subscale may be consistent with observations that γ-band activity is sensitive to perception of speech sounds and lexicality [70-72]. Frontal γ-band EEG power is strongly correlated with expressive and receptive language skill in 24 to 36-month-old children [73]. The observed inverse relationship between SRS scores and tGBR and ASSR γ-band evoked power suggests that there may be a relationship between γ-band dysfunction and important traits in ASD such as social skill, although it seems unlikely that passive auditory γ-band power is directly related in any causal manner to social reciprocity. More likely, the auditory findings we describe are related to those found in other areas of the cerebral cortex that are more directly related to social cognitive function. For example, previous studies have shown γ-band changes in ASD in the context of face perception and eye-gaze processing [37,74].

It is worth emphasizing that the pASD sample in this study did not exhibit a strong presentation of ASD/BAP traits based on the results of the AQ and SRS data. Group differences were found only for the attention to local detail subtest of the AQ. It is possible that this was due to the use of singleton families in the study, as previous research has indicated that the BAP is expressed more strongly in multiplex autism families [6,75]. Nonetheless, it is important that we found differences in a biological marker in the singleton families because simple biomarkers may be more sensitive than behavioral phenotypes to risk for ASD. A future study might usefully compare single- and multiple-incidence family members to assess whether the multiple-incidence families or those who express the BAP strongly exhibit stronger γ-band findings than the single-incidence families. Furthermore, because the number of fathers in the pASD sample was low relative to mothers, it is possible that increasing the number of men in future studies would reveal differences based on parent gender, as some BAP studies have suggested that it is more strongly expressed in fathers than mothers [76]

## Conclusions

The findings of reduced phase-locking and evoked γ-band power in first-degree relatives of people with ASD are consistent with a heritable neural synchrony endophenotype. In the current study, ASSR γ-band measures were significantly different between groups, whereas tGBR measures were not, suggesting that the ASSR may be a more robust measure in first-degree relatives. Although the findings in this study are consistent with heritability, heritability itself was not directly measured in this study and, strictly speaking, the group differences observed in this study should be considered evidence for familiality. Family studies, particularly twin designs, would be necessary to provide direct measures of heritability. A caveat to these findings is that reduced phase-locking and evoked power in the γ-band range is not specific to ASD. Similar, if not identical, deficits occur in people with schizophrenia and their first-degree relatives [77-79]. This limits the utility of the finding as a diagnostic biomarker. However, γ-band measures markers should be useful in genetics studies and as biomarkers of drug response in future clinical trials. Future research should focus on establishment of a normal range for each γ-band measure in healthy subjects, definition of useful cut-offs for abnormal values, and the relationship between γ-band markers and measures of cortical GABA and glutamate concentrations in people with ASD and in animal models of ASD.

## Methods

### Ethics

Participants signed informed consent to participate in the experiment, consistent with the guidelines of the Colorado Multiple Institution Review Board.

### Subjects

Parents of children with an autism spectrum disorder (pASD: 6 men, 15 women) participated in the study. Each parent had a child who met the *Diagnostic and Statistical Manual*, fourth edition (DSM-IV) criteria for an autism spectrum disorder (Autistic disorder or Asperger's syndrome), as determined by consensus of the Autism Diagnostic Observation Schedule [80], the Autism Diagnostic Interview, Revised [ADI-R: [81]] and DSM-IV diagnosis by an experienced clinical psychologist (SH). Of the twenty-one probands, seventeen were male and four were female. Twenty adults (seven men, thirteen women) with no personal or family history of pervasive developmental disorder were recruited to serve as healthy comparison (HC) subjects. All participants were tested for hearing thresholds using the method of constant stimuli and did not exceed 20 dB HL for the stimulation frequency used in the experiment.

### Behavioral measures

The Wechsler Abbreviated Scale of Intelligence (WASI) [82] was used to assess general cognitive function. The AQ [41] is a self-administered scale of autism symptoms that includes five subscales: 1) communication, 2) social skills, 3) imagination, 4) attention to detail and 5) attention switching. Total AQ scores range from 0 to 50, with higher scores more indicative of traits associated with autism. The Social Responsiveness Scale (SRS) [42] is an informant-based measure of reciprocal social behavior, and was also given to all participants' live-in spouse or partner for rating. Scores on the SRS range from 0 to 195, with higher scores indicating more problems with social reciprocity. The Hollingshead four-factor index of social position [83] was calculated as a measure of socioeconomic status (SES), with higher values indicating higher SES. Table 1 contains mean and standard deviation information for all demographic and phenotype variables assessed.

### Auditory stimuli

MEG recordings were made to binaural presentations of amplitude-modulated (AM) white-noise stimuli (16-bit quantization, 500 ms duration, 100% AM depth, 75 dB sound-pressure limit at the ear). Three AM frequencies were used in separate blocks: 32, 40 and 48 Hz. Stimuli were delivered via foam insert earphones (E.A.R., Cabot Safety Co., Indianapolis, IN, USA). In total, 150 discrete stimulus trials with a 2 second inter-stimulus interval were delivered per AM frequency block.

### MEG procedures

MEG data were acquired with a brain imaging unit (Magnes WH3600; 4D Neuroimaging, San Diego, CA, USA) with 248 axial first-order gradiometers inside a magnetically shielded room. Participants were recorded supine, and were allowed to view a silent video of their choice during recordings. MEG data were continuously acquired at 24-bits quantization and sampling rate of 678.1 Hz, using a pass band of 0.1 and 200 Hz.

The location and orientation of the MEG coils relative to each subject's head were determined before recording by digitizing fiducial reference points on the head using a magnetic digitizer (Polhemus 3SPACE, Colchester, VT, USA). The left and right preauricular points and the nasion were used to establish a right-handed Cartesian coordinate system, where the line between left and right preauriculars is the *x*-axis with positive *x *exiting out the left ear. The *y*-axis is the line normal to the *x*-axis at the midpoint (origin), with positive *y *exiting through the front of the head at the nasion, and the *z*-axis is normal to *x *and *y *at the origin with positive *z *exiting at the top of the head. After digitizing the reference points, the shape of each subject's head under the recording surface of the MEG system was digitized between 3000 and 5000 points for use in constructing a volume conductor model for MEG source localizations.

### Data pre-processing and source modelling

Epochs of -200 to 800 ms were defined around the stimulus onset. All data epochs with values exceeding ± 2000 fT were rejected from further analysis to exclude trials with non-physiological artifacts (for example,, movement, eye blinks). Noisy or otherwise compromised channels (that is, those whose values consistently exceeded 2000 fT or were less than 50 fT during recording) were removed from analysis. Remaining epochs were visually inspected for any other artifacts, which were marked and removed, and then grand averaged across AM conditions to produce averages for source analysis. Epochs marked as containing artifact were not used in any other subsequent analyses. Averages were baseline corrected using the pre-stimulus period (-0.2 to 0 seconds) and were digitally low-pass filtered (24 dB/octave, phase invariant Butterworth) at 20 Hz. This low-pass filter setting was only applied to the grand averaged data for source analysis.

Source analyses of the grand-averaged data for each subject (mean ± SD trials: HC 400.33 ± 67.52, pASD: 384.10 ± 75.55; *t*_(37) _= 0.71; *P *> 0.4) were conducted using the 4DNeuroimaging software. We conducted the source analysis on the early, M100 averaged evoked response, rather than the tGBR or ASSR responses, for two reasons: 1) we hypothesized group differences in γ-band activity and did not wish to perform the source analysis using inverse solutions whose goodness of fit would probably differ between groups, and 2) we wanted to grand average the three conditions, each of which produced the M100 response, to control for differences in signal-to-noise ratio that could have systematic effects on the time-frequency estimates if different source models had been used for each AM condition independently. This procedure was designed to reduce the effect that location, particularly depth, might exert on the dipole moment, by fixing the location for each condition according to the overall grand average fit. It is worth noting that source orientation is similar between the γ-band response and the M100 and the small difference in spatial locations has minimal effects on the dipole waveform data (see next section), because of the inherently high lead field correlation between closely adjacent sources. Equivalent current dipoles (ECD) were fitted for the left and right hemispheres in the post-stimulus window between 60 and120 ms, yielding parameter estimates of the *x*, *y *and *z *ECD position information and the dipole orientation and magnitude over time. The M100 component fit selected for subsequent analyses corresponded with the best-fitting time point between 60 and 120 ms with a negative z axis current component and residual model error less than or equal to 10%.

### Source space projection and time-frequency analysis

**Figure 4:**
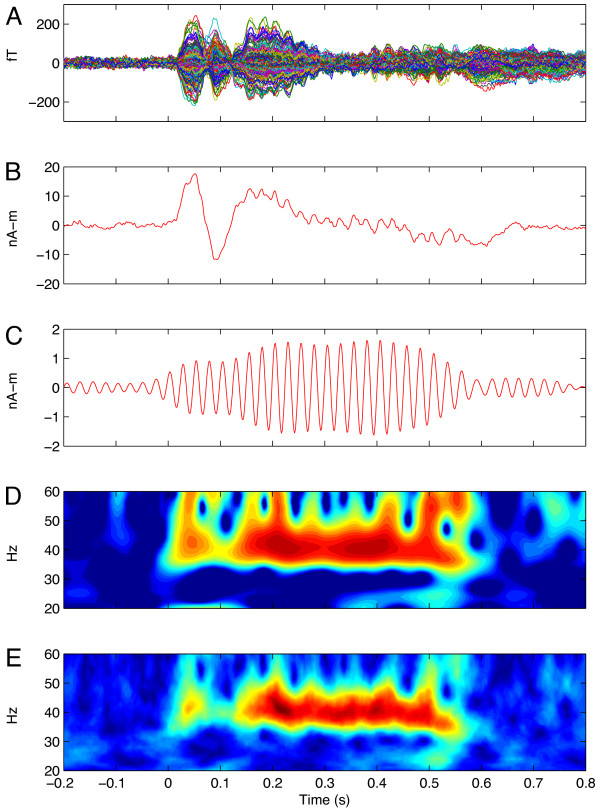
**Data example**. **(A) **Unfiltered time-domain average 248-sensor waveforms from single subject. **(B) **Unfiltered, averaged dipole waveform for right auditory cortex corresponding to data in A. **(C) **Waveform in B filtered using a 35 to 45 Hz bandpass to emphasize γ-band in time-domain (not part of data analysis). **(D) **Time-frequency representation of phase-locked, baseline-normalized evoked power from same waveform in (B). **(E) **Time-frequency representation of phase-locking factor (PLF) corresponding to data in (B).

The ECD parameters from the dipole fits were then saved and used to project the epoched, artifact-free, 248-channel MEG time series into source space using source space projection (SSP, also referred to as signal space projection [84]). SSP is an inverse-spatial filter approach that results in a significantly reduced dataset in source, rather than sensor, space (that i,, dipole waveforms, sometimes referred to as 'virtual electrodes'). The SSP channel time series for the left and right hemispheres, Q_(t)_, in amplitude units of nA-m, were then transformed using the Morlet wavelet decomposition [85]. Details of this procedure can be found in Teale *et al*. [48]. Briefly, the individual trials of the source waveforms were convolved with wavelets (wave number 7) in 1 Hz increments from 20 to 60 Hz. The modulus of the amplitude-normalized mean across trials at each sample point is then taken as the phase-locking factor (PLF), whose value varies from 0 (random phase) to 1 (perfect phase-locking). PLF represents the inter-trial phase-consistency and is also referred to as inter-trial coherence [86,87]. The evoked (phase-locked) and induced (non-phase-locked) source power between 20 and 60 Hz were also calculated and expressed in dB units of change relative to the pre-stimulus baseline (-0.2 to 0 seconds). Total power (evoked + induced) relative to baseline was also calculated. The PLF, evoked, induced and total power metrics were calculated for each of the three conditions and two hemispheres. Figure 4 provides examples of the various waveforms and time-frequency comparisons used in the study. Baseline power and peak frequency were also extracted for statistical comparison. To examine the type of γ-band (transient versus steady-state), the mean of each variable within two time-frequency windows of interest was computed: 1) from 30 to 100 ms post-stimulus and 2) from 200-500 ms post stimulus. For each modulation condition in the ASSR window, the frequency of interest was a 9-Hz window centered on the modulation frequency (that is, modulation frequency ± 4 Hz). For tGBR, the frequency window was fixed between 30 and 50 Hz because, in contrast to ASSR, the tGBR response frequency is expected to be independent of the AM frequency. All time-frequency computations were conducted using our own custom routines written using MATLAB (version 2009b; MathWorks, Inc., Natick, MA, USA). Figure 3 illustrates key output variables for a single subject's MEG data.

Statistical analyses were conducted within SPSS (version 18, SPSS Inc., Chicago, IL, USA). For ANOVA/MANOVA designs, type III sums of squares were used. Pearson *r *correlation coefficients were computed between BAP and other demographic measures and time-frequency measures. To reduce the number of comparisons for the correlations, we used mean PLF, baselines and baseline-normalized power across modulation frequency for each hemisphere (for example, left 32, 40 and 48 Hz PLF were averaged together).

## List of abbreviations

AM: amplitude modulation; AQ: Autism-Spectrum Quotient; ASSR: auditory steady-state response; BAP: broad autism phenotype (BAP); GABA: Gamma-amino butyric acid; GAD: glutamate decarboxylase; HC: healthy control; MEG: magnetoencephalography; pASD: parents of children with autism spectrum disorder; PLF: phase-locking factor; SSP: source space projection; SRS: social responsiveness scale; tGBR: transient γ-band response; WASI: Wechsler Abbreviated Scale of Intelligence.

## Competing interests

The authors declare that they have no competing interests.

## Authors' contributions

DR designed the study, carried out the final statistical analyses and drafted the manuscript. KY, LW and AW assisted with study coordination, administration and scoring of behavioural measures. PT, EK and KM provided key input on MEG data analysis, and wrote most of the customized routines used in the analysis of data. SH provided input on behavioral measure selection and scoring, and was involved in the clinical diagnostic evaluation of probands. All authors read and approved the final manuscript.

## Supplementary Material

Additional file 1 **Simulation of effects of added phase and amplitude noise on evoked power and phase-locking factor**. Additional File 1 contains methods, results and a figure relating to simulation of the effects of adding phase and amplitude noise to a known signal, such that the effect of noise on measures of evoked power and phase-locking can be evaluated. Click here for file
