# Supplementary material for: Transient and steady-state auditory gamma-band responses in first-degree relatives of people with autism spectrum disorder
Source: Mol Autism. 2011 Jul 5;2:11. doi: 10.1186/2040-2392-2-11 (PMC3143088; doi:10.1186/2040-2392-2-11)
Supplement: Additional file 1 — Simulation of effects of added phase and amplitude noise on evoked power and phase-locking factor. Additional File 1 contains methods, results and a figure relating to simulation of the effects of adding phase and amplitude noise to a known signal, such that the effect of noise on measures of evoked power and phase-locking can be evaluated. [file 2040-2392-2-11-S1.DOC]

## Supplemental Data and Figure

To address reviewer concern over the concept that adding noise to signal will affect the amplitude-based measure of power more than the amplitude independent measure of PLF, we performed a simulation, presented here as supplemental data. Data for the simulation were 100 trials of sine wave stimuli (40 Hz) sampled at 1 kHz. Three parameters were systematically varied: 1) the amount of phase-variability from trial to trial (from low to high: π/8, π/4, 3*π/8, π/2, 5*π/8, 3*π/4, 7*π/8 and π), 2) the amount of amplitude variability from trial to trial, and 3) whether or not additional, random noise was present (signal to noise ratio = 1:1). The epoch duration for the trials was 2 seconds.

The effect on variance across trials is illustrated in Supplemental Figure 1. Note that for PLF, while increasing inter-trial phase-variability clearly decreases the PLF, amplitude variance and additional random noise have no visible effect. However, for evoked amplitude, both increasing amplitude variability in the signal, as well as added random noise, result in lower amplitudes and more inter-trial variability. In the lowest 4 phase-variability conditions, in the presence of no added noise (see Figure 1C), evoked amplitudes reflect phase-locking reasonably well. This supports our contention, which is common in the literature, that measures of phase-variability are more resistant to increased noise in the data than are measures that are not amplitude independent.

## Supplemental Figure 1. Effects of varying phase, amplitude and additional noise on phase-locking factor and spectral amplitude
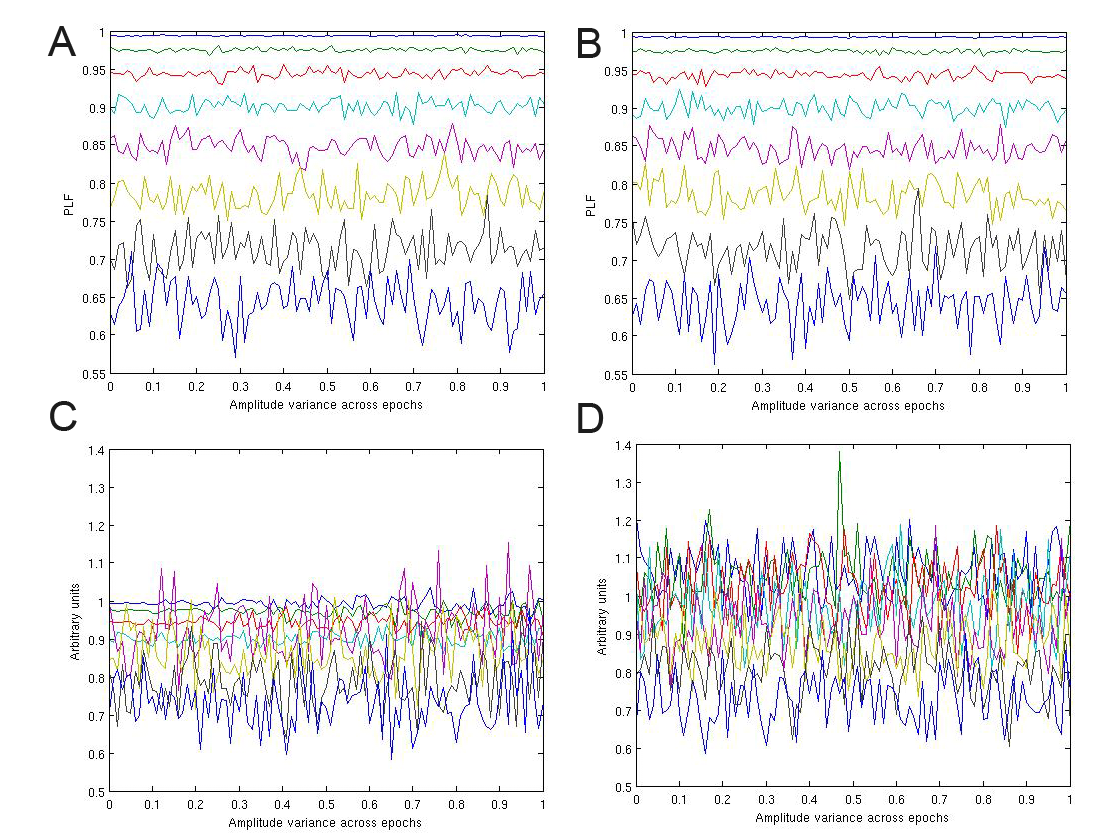


Supplemental Figure 1 caption: In these four panels, inter-trial amplitude variability in the 40 Hz signal increases from left to right along the horizontal axis (0 equals no variance across trials, and for any value of x, amplitude was uniformly distributed between 1 – x/2 and 1 + x/2). On the vertical axes, the measure is either PLF (A and B) or amplitude in arbitrary units (C and D). Each point represents the mean of 100 trials. The no additional noise condition is shown in the panels on the left (A and C) and the additional random noise condition is shown in the panels on the right (B and D). The colors indicate the phase-variability conditions (dark blue = π/8, green = π/4, red = 3*π/8, cyan = π/2, purple = 5*π/8, yellow = 3*π/4, black = 7*π/8, light blue = π).
